# Testing of a MEMS Dynamic Inclinometer Using the Stewart Platform

**DOI:** 10.3390/s19194233

**Published:** 2019-09-29

**Authors:** Zhihua Liu, Chenguang Cai, Ming Yang, Ying Zhang

**Affiliations:** 1 Division of Mechanics and Acoustics, National Institute of Metrology of China, Beijing 100029, China; caichenguang@nim.ac.cn (C.C.); zhangying171@mails.ucas.edu.cn (Y.Z.); 2 College of Information Science and Technology, Beijing University of Chemical Technology, Beijing 100029, China; Yang_M1230@yeah.net

**Keywords:** MEMS inclinometer, dynamic performance, spatial orbit, Stewart platform

## Abstract

The micro-electro-mechanical system (MEMS) dynamic inclinometer integrates a tri-axis gyroscope and a tri-axis accelerometer for real-time tilt measurement. The Stewart platform has the ability to generate six degrees of freedom of spatial orbits. The method of applying spatial orbits to the testing of MEMS inclinometers is investigated. Inverse and forward kinematics are analyzed for controlling and measuring the position and orientation of the Stewart platform. The Stewart platform is controlled to generate a conical motion, based on which the sensitivities of the gyroscope, accelerometer, and tilt sensing are determined. Spatial positional orbits are also generated in order to obtain the tilt angles caused by the cross-coupling influence. The experiment is conducted to show that the tested amplitude frequency deviations of the gyroscope and tilt sensing sensitivities between the Stewart platform and the traditional rotator are less than 0.2 dB and 0.1 dB, respectively.

## 1. Introduction

Thanks to recent advances in micro-electro-mechanical-system (MEMS) technology, the size of the sensor can be dramatically reduced to the chip size, along with reductions in cost and power consumption [1,2]. Inclinometer sensors for tilt measurements have been widely applied in many industrial applications, such as structural health monitoring, ground movement measurement, and attitude dynamic measurement and control [3,4,5,6].

The MEMS dynamic inclinometer integrates a tri-axis gyroscope and a tri-axis accelerometer for real-time tilt measurement [7,8]. As the data from the accelerometer are in general very noisy and susceptible to external acceleration interference, a gyroscope offers unsusceptible angular velocities around the axes. The data from a gyroscope have a tendency to drift, because of the angular velocity data bias accumulation over time; therefore, the data fusion of the gyroscope and accelerometer is a complete solution for the dynamic tilt measurement.

The MEMS sensor must be calibrated before being used and re-calibrated periodically for precision applications. The six-position static test and the rate test are among the most commonly used methods for the calibration of MEMS accelerometers and gyroscopes [9,10]. The six-position method requires a perfect cube shaped mounting frame to make each sensitive axis of the accelerometer point alternatively up and down. To estimate the axis misalignments, an improved six-position test can be performed, which takes into account bias, scale factors, and non-orthogonalities. The rate test is typically done using a precision rate turntable by rotating the gyroscope through the given turning rates, and comparing them with the outputs of the gyroscope. A multi-axis turntable is often used for the IMU calibration by making use of gravity and Earth rotation rates as references. This method is not applicable to MEMS gyroscopes, as the Earth’s rotation is a very weak signal that is typically buried within the sensor noise.

The current methods for MEMS sensors are applicable for calibrating the error models of MEMS inclinometers, including scale factors, bias, and non-orthogonalities [11,12,13,14]. Less attention has been paid to the dynamic performance of the MEMS inclinometer. To evaluate the tracking performance and cross-coupling of MEMS dynamic inclinometers, a testing device for multiple degrees of freedom of tilt motion and positional motion is required. The Stewart platform consists of two plates jointed by six prismatic actuators that allow it to be precisely adjusted and controlled [15,16]. Devices placed on the top plate can be moved in six degrees of freedom, as follows: Three linear movements and three rotations [17]. Spatial orbits that are generated by the Stewart platform have an advantage over uniaxial motion in dynamic performance testing for MEMS inclinometers.

This paper performs research on a new approach for testing a MEMS dynamic inclinometer by using a Stewart platform. The paper is organized as follows: Section 2 introduces the kinematic analysis and spatial orbits of the Stewart platform for testing purpose; Section 3 discusses the testing method for the MEMS inclinometer using spatial orbits; Section 4 presents experimental investigations on the tracking performance and cross-coupling influence, and a comparison with the rotator; and the last section provides conclusions of the paper.

## 2. Stewart Platform

### 2.1. Kinematic Analysis

**Figure 1:**
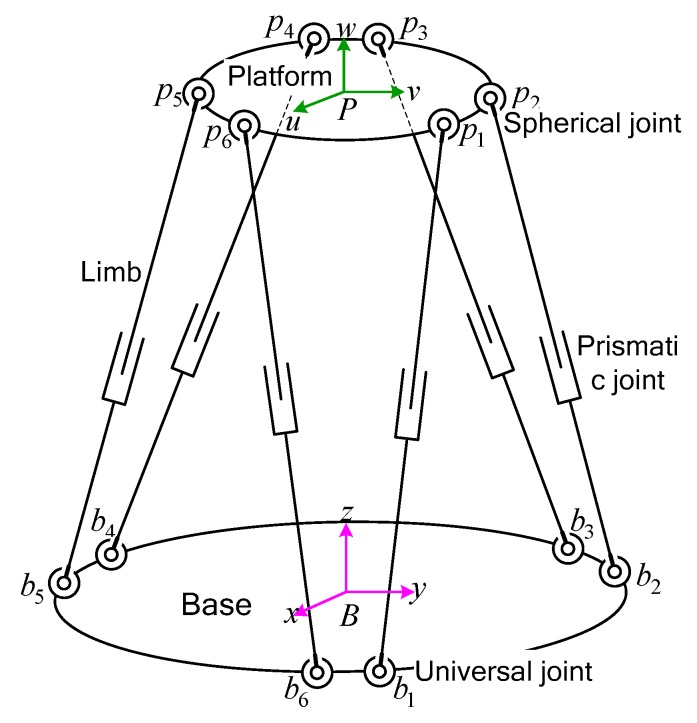
Schematic view of the Stewart platform.

As shown in Figure 1, the Stewart platform consists of a fixed base, a moving platform, and six limbs. The limbs are composed of two parts, an upper link and a lower link, connected by a prismatic joint. The upper link connects the moving platform using a spherical joint, and the lower link connects the fixed base using a universal joint. A servo motor via a linear ball screw is used to change the length of each limb so as to generate the position and orientation of the moving platform.

**Figure 2:**
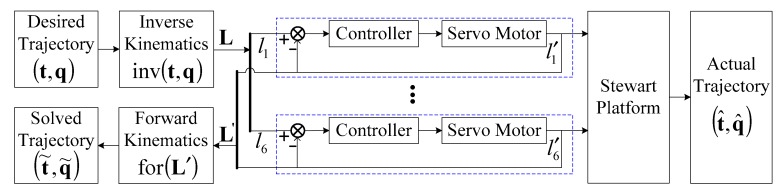
Control and measure of the position and orientation of the Stewart platform.

Let (t,q) stand for the position and the orientation of the moving platform, where q is the quaternion defined by a single rotation about a vector. Inverse kinematics is implemented for the control of the Stewart platform [18]. As shown in Figure 2, the length of the ith limb, $l_{i}$, is calculated from the desired trajectory of the position and the orientation (t,q). The servo motors are controlled to drive the limbs to follow the corresponding calculated lengths. However, amplitude attenuation and phase lag exist between the actual lengths, $l_{i}^{\prime }$, and the calculated lengths, $l_{i}$, between the characteristics of the closed-loop frequency responses of the servo motors [19,20]. The actual trajectory $\left(\hat{t},\hat{q}\right)$ is generated on the moving platform by the six extension limbs, and is further used for testing.

The Stewart platform that was used to test the MEMS inclinometer was calibrated in advance, and all of the kinematics parameters, including the length offsets of the limbs, positions of the spherical joints, and positions of the universal joints, were identified and compensated. The calibration was implemented by means of extra measurements, such as by a laser tracker or a bar ball, to compare with the internal encoders of the mechanism [21]. Therefore, it is acceptable to measure the position and orientation of the platform by using the internal encoders, as the kinematics parameters were compensated after the calibration.

As there may be deviation between the desired trajectory and the actual trajectory, forward kinematics is implemented to get more accurate trajectories of the position and orientation $\left(\tilde{t},\tilde{q}\right)$ from the six actual limb lengths, $L^{\prime }={\left[l_{1}^{\prime },\cdots l_{6}^{\prime }\right]}^{T}$. The position and orientation $\left(\tilde{t},\tilde{q}\right)$ can be solved by the following nonlinear equations [22].

### 2.2. Spatial Orbits

**Figure 3:**
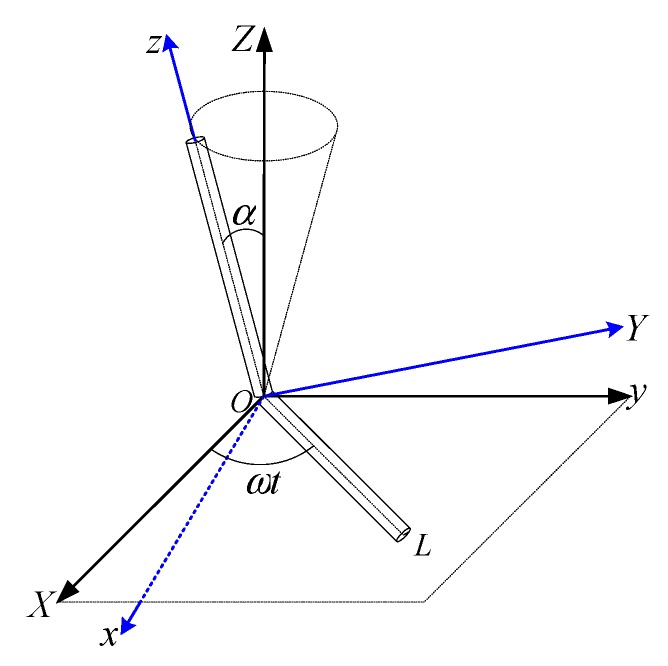
Conical motion around the Z-axis.

The spatial orbits generated by the Stewart platform can be applied for the testing of the MEMS dynamic inclinometer. The conical motion around the Z-axis is generated to test the tracking performance of the inclinometer. As illustrated in Figure 3, the conical motion around the Z-axis is formed by a single rotation about a vector, OL, through an angle, and the vector, OL, rotates at a constant angular velocity in the XOY plane. The attitude representation in terms of the quaternion is described as follows:

(1) $$q\left(t\right)={\left(\begin{array}{cccc} \cos\frac{\alpha }{2} & \sin\frac{\alpha }{2}\cos\omega t & \sin\frac{\alpha }{2}\sin\omega t & 0 \end{array}\right)}^{T}$$

where α is the constant rotation angle and ω is the angular velocity of vector, OL. The conical motion has advantages over the one-dimensional rotation motion in dynamic tilt testing, because first, it provides two degrees of freedom of tilt motion, that is, cyclic symmetry, and second, it is a kind of dynamic orbit with sinusoidal components of a specific frequency.

The angular velocity of the conical motion around the Z-axis is given by the following:

(2) $$\omega \left(t\right)={\left(\begin{array}{ccc} -\omega \sin\alpha \sin\omega t & \omega \sin\alpha \cos\omega t & -2\omega \sin^{2}\frac{\alpha }{2} \end{array}\right)}^{T}$$

Here, the X- and Y-axis components of the angular velocity both have simple harmonic motion, which can be used to test the gyroscope mounted on the inclinometer, while the Z-axis component is just a small constant value, so that is not applicable for testing.

Furthermore, according to the authors of [23], the acceleration components along the three axes due to the tilt angles in the Earth gravity field are derived as follows

(3) $$\left(\begin{array}{c} a_{x}/g\\ a_{y}/g\\ a_{z}/g \end{array}\right)=R{\left(q\right)}^{T}\left(\begin{array}{c} 0\\ 0\\ 1 \end{array}\right)=\left(\begin{array}{r} -\sin\alpha \sin\omega t\\ \sin\alpha \cos\omega t\\ \cos\alpha \end{array}\right)$$

where R(q) is the rotation matrix expressed in terms of the quaternion; $a_{x}$, $a_{y}$, and $a_{z}$ are the acceleration components; and g is the local gravity. Here, the harmonic motion of the X- and Y-axes is useful for testing the accelerometer mounted on the inclinometer.

**Figure 4:**
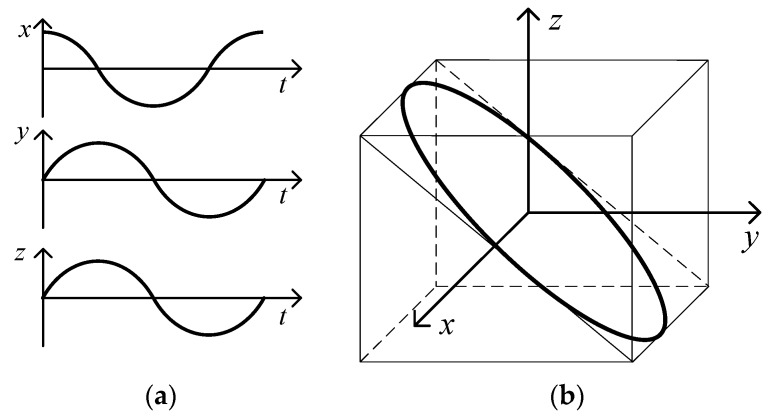
Positional orbit. (**a**) Time–domain waveform; (**b**) Spatial elliptical orbit.

Besides the conical motion for the angular motion excitation, the positional motion is generated to test the cross coupling influence of the inclinometer. As discussed in the literature [24], harmonic positional vibrations along the three orthogonal axes with the same frequency are bound to composite a spatial positional elliptical orbit, as depicted in Figure 4; the shape and orientation depend on the amplitudes and phases of the harmonic vibrations. The three orthogonal positional vibrations at a specified angular frequency, ω, can be written as follows:

(4) $$S_{i}\left(t\right)={\hat{S}}_{i}\sin\left(\omega t+\phi_{i}\right)\;i\in \left\{x,y,z\right\}$$

where ${\hat{S}}_{i}$ is the amplitude of the positional vibration and $\phi_{i}$ is for the phase.

## 3. Testing Using Spatial Orbits

### 3.1. Gyroscope and Accelerometer

**Figure 5:**
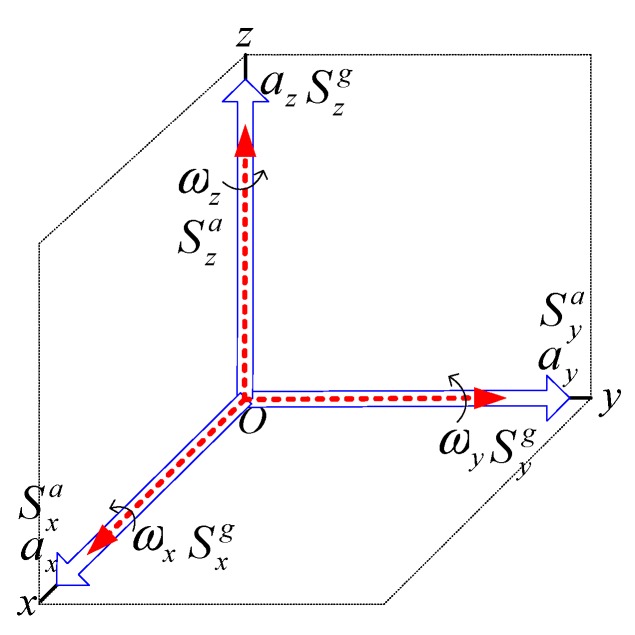
Sensitivity axes of the gyroscope and accelerometer.

The MEMS dynamic inclinometer consists of a tri-axis gyroscope and a tri-axis accelerometer for real time tilt sensing. The gyroscope and the accelerometer give six degrees of freedom capable of measuring the angular velocity around the three axes and the acceleration along the three axes. However, because of the imprecision in the construction of the inclinometer, the three axes of the gyroscope and the accelerometer form two distinct non-orthogonal frames. Let us define a reference coordinate frame on the inclinometer. Both the gyroscope and accelerometer frames are unaligned with the reference coordinate frame. Calibration is implemented by manufacturers to transform the non-orthogonal gyroscope and accelerometer sensitivity axes into the reference orthogonal coordinate frame. Then, the inclinometer output is proportional to the physical quantities sensed by the accelerations and the angular rates, respectively. As shown in Figure 5, the relationship between the output and the physical quantity acting along the reference coordinate frame can be expressed by a diagonal sensitivity matrix

(5) $$S^{j}=\mathrm{diag}\left(\begin{array}{ccc} S_{x}^{j} & S_{y}^{j} & S_{z}^{j} \end{array}\right)\;j\in \left\{a,g\right\}$$

where $S_{x}^{j}$, $S_{y}^{j}$, and $S_{z}^{j}$ are the X-, Y-, and Z-axial sensitivities, respectively; j=g is the gyroscope case; and j=a is the accelerometer case.

For the gyroscope case, from Equation (2), we can get the X- and Y-axial sensitivities as follows:

(6) $$\left\{ \begin{array}{l} S_{x}^{g}=Y_{x}^{g}/\left(\omega \sin\alpha \right)\\ S_{y}^{g}=Y_{y}^{g}/\left(\omega \sin\alpha \right) \end{array}\right.$$

where $Y_{x}^{g}$ and $Y_{y}^{g}$ are the X- and Y-axial amplitudes of the gyroscope output under the excitation of the conical motion around the Z-axis, and α and ω are the conical angle and angular frequency of the conical motion generated by the Stewart platform, respectively. The Z-axial sensitivity, $S_{z}^{g}$, needs to be determined through the conical motion around the X- or Y-axis.

For the accelerometer case, from Equation (3) we can get the X- and Y-axis sensitivities as

(7) $$\left\{ \begin{array}{l} S_{x}^{a}=Y_{x}^{a}/\left(g\sin\alpha \right)\\ S_{y}^{a}=Y_{y}^{a}/\left(g\sin\alpha \right) \end{array}\right.$$

where $Y_{x}^{a}$ and $Y_{y}^{a}$ are the X- and Y-axial amplitudes of the accelerometer output under the excitation of conical motion around the Z-axis, and α is the conical angle of the conical motion generated by the Stewart platform. The difference here is that it is impossible to be obtain the Z-axial sensitivity, $S_{z}^{a}$, through other conical motions, unless the inclinometer is re-installed so that its Z-axis is horizontal.

### 3.2. Tilt Sensing

The tilt of the sensor is usually defined in terms of the roll angle, φ, and the pitch angle, θ, while the yaw angle, ψ, is not provided. The orientation is determined by both the rotation angles and the order in which these rotations are to be applied. The angle set ${\left(\begin{array}{ccc} \varphi & \theta & \psi \end{array}\right)}^{T}$ is not a completely informative vector as the rotation order is still needed to express the orientation. To be unaffected by the rotation order of the orientation angles, the sensitivity needs to be defined on the condition that only one orientation angle input is active and the other two are zero. However, this kind of angular sensitivity is deficient if multiple orientation angles are active at the same time.

Therefore, we define the sensitivities based on a quaternion that has nothing to with the rotation order. If the conical motion around the Z-axis is acted on the inclinometer, the desired tilt output in terms of the quaternion should match with Equation (1), based upon which the sensitivities are given by the following

(8) $$\left\{ \begin{array}{l} S_{x}^{C}=Y_{x}^{C}/\left(\sin\frac{\alpha }{2}\right)\\ S_{y}^{C}=Y_{y}^{C}/\left(\sin\frac{\alpha }{2}\right) \end{array}\right.$$

where $Y_{x}^{C}$ and $Y_{y}^{C}$ are the amplitudes of the X- and Y-axial components of the rotation vector in the quaternion, and α is the conical angle of the conical motion generated by the Stewart platform.

If the roll and pitch angles are offered by the inclinometer, the quaternion output needs to be derived first. The last column of the rotation matrix formulated by the rotation angles at any orientation is given by the following:

(9) $$R{\left(\varphi ,\theta ,\psi \right)}^{T}\left(\begin{array}{c} 0\\ 0\\ 1 \end{array}\right)=\left(\begin{array}{r} -\cos\varphi \sin\theta \\ \sin\varphi \\ \cos\varphi \cos\theta \end{array}\right)$$

where R(φ,θ,ψ) is the rotation matrix expressed in terms of the Euler angles.

By comparison with the angle formulated by the quaternion in Equation (3), we can derive the rotation angles for the conical motion around the Z-axis as follows:

(10) $$\left\{ \begin{array}{c} \sin\varphi =\sin\alpha \cos\omega t\\ \tan\theta =\tan\alpha \sin\omega t \end{array}\right.$$

Equation (10) describes that the sine of roll angle, φ, and the tangent of pitch angle, θ, are both harmonic vibrations with amplitudes of sinα and tanα, respectively. If we take ${\left(\begin{array}{cc} \sin\varphi & \tan\theta \end{array}\right)}^{T}$ as the tilt outputs, we can define the following sensitivities as follows:

(11) $$\left\{ \begin{array}{c} S_{s\varphi }^{C}=Y_{s\varphi }^{C}/\sin\alpha \\ S_{t\theta }^{C}=Y_{t\theta }^{C}/\tan\alpha \end{array}\right.$$

where $Y_{s\varphi }^{C}$ and $Y_{t\theta }^{C}$ are the amplitudes of the tilt output ${\left(\begin{array}{cc} \sin\varphi & \tan\theta \end{array}\right)}^{T}$.

The dynamic inclinometer offers real-time tilt measurement by data fusion from the gyroscope and the accelerometer. The positional motion may have an influence on the tilt measurement of an inclinometer because of the produced acceleration. The tilt output resulting from the positional motion is the cross coupling that it is hoped to be reduced to minimum. Let us define the cross-coupling of an inclinometer as the maximum tilt angles under the action of a spatial positional orbit generated by the Stewart platform.

## 4. Experimental Investigation

**Figure 6:**
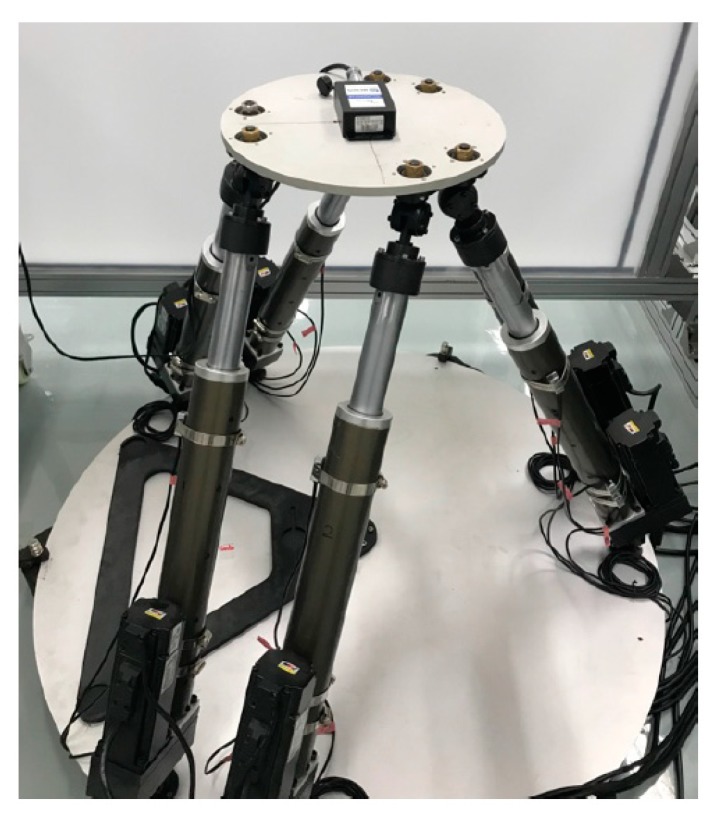
The Stewart platform for the MEMS inclinometer testing.

In this experimental investigation, we applied the Stewart platform to generate spatial orbits for the testing of the MEMS inclinometer. As shown in Figure 6, the MEMS inclinometer was mounted with its sensitivity axes parallel to the reference coordinate frame of the moving platform. The characteristics of the MEMS inclinometer (model number: BW-VG 527) are provided in Table 1.

The lengths of the six limbs are calculated from the desired spatial orbits by implementing inverse kinematics. The servo motors are precisely controlled to dynamically follow the calculated lengths. The outputs of the MEMS inclinometer that respond to the spatial orbit are acquired and in the meantime, the actual lengths of the six limbs are measured through the encoders of the servo motors. Forward kinematics is implemented to solve the position and orientation of the moving platform, by comparing which can be used to test the MEMS inclinometer.

### 4.1. Tracking Performance

**Figure 7:**
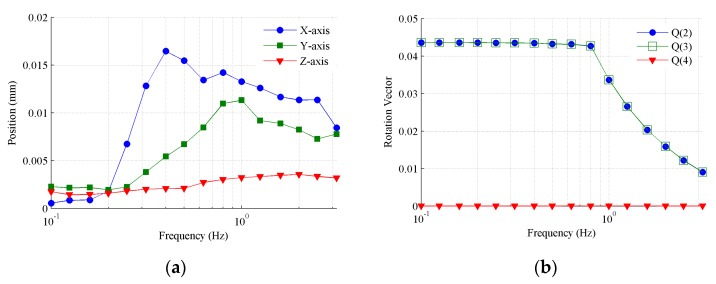
Conical motion around the Z-axis generated by the Stewart platform. (**a**) Amplitudes of position; (**b**) amplitudes of orientation where the legend Q(i) (i = 2, 3, 4) stands for the ith element of the quaternion for the orientation expression.

The conical motion around the Z-axis, with various frequencies from 0.1 Hz to 3.15 Hz, was first generated as the spatial motion excitation to test the tracking performance of the MEMS inclinometer. A constant rotation angle of 5° was selected as the desired command at low frequencies, while in the case of exceeding the maximum acceleration capacity of the Stewart platform, gradually declining rotation angles were selected from 1 Hz to 3.15 Hz. Figure 7 shows the amplitudes of the orientation and position that are solved from the measured encoders. As shown in Figure 7a, the amplitudes of the position are less than 0.017 mm over the whole frequency range, so we can neglect their influence. Figure 7b shows the amplitudes of the orientation in terms of the quaternion elements. According to Equation (6), the second and third quaternion elements are both simple harmonic signals with a specific amplitude, while the fourth quaternion element is a constant zero. As shown in Figure 7b, the X- and Y-axis components of the quaternion elements have a nearly identical amplitude, and the amplitude of the Z-axis component is far less than the other two. This shows that the generated conical motions around the Z-axis are well qualified for testing purposes.

**Figure 8:**
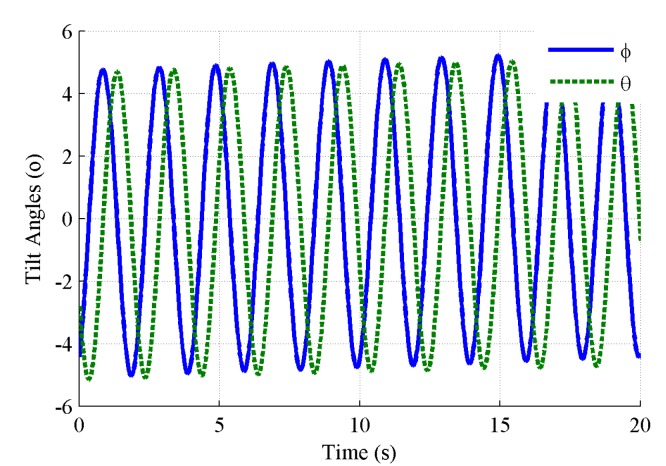
Tilt angles of the MEMS inclinometer at a conical motion around the Z-axis.

The outputs of the tilt angles at the conical motion around the Z-axis with 0.5 Hz are depicted in Figure 8. There are obvious trend components in the period tilt angles because of integration errors. Note that the waveforms are very close to, but not identical to, simple harmonic signals, while the sine of the roll angle and the tangent of the pitch angles are simple harmonic signals according to Equation (10).

**Figure 9:**
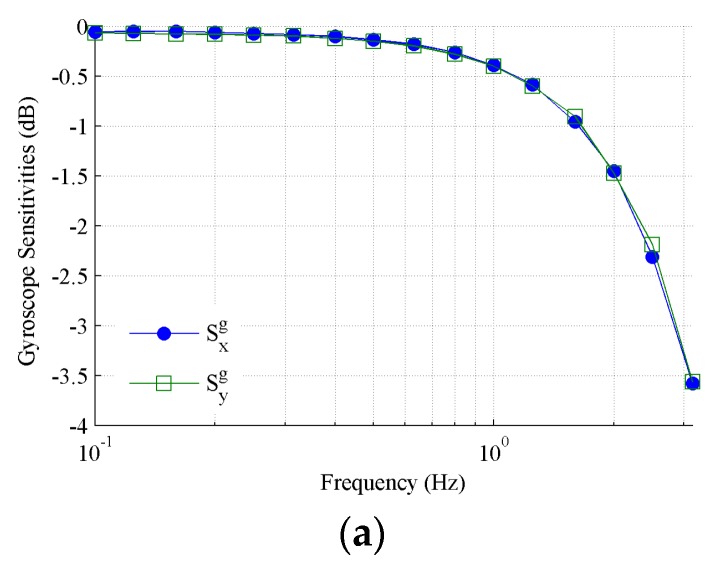

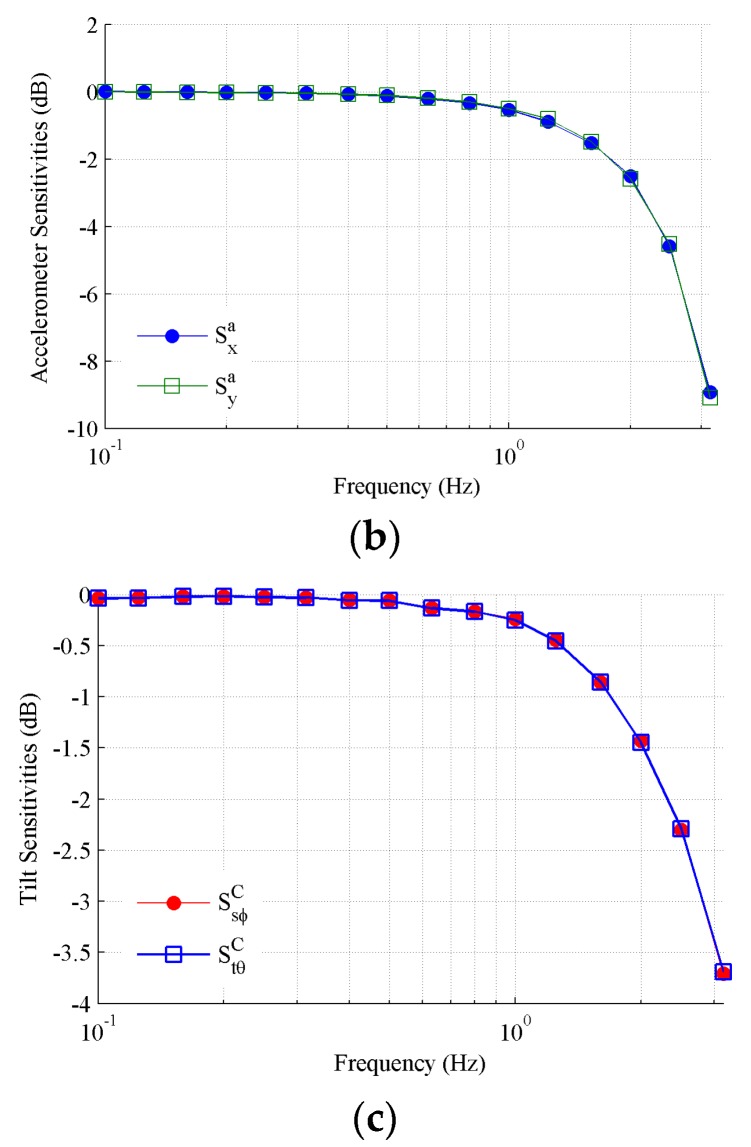
Amplitude frequency curves. (**a**) Accelerometer; (**b**) gyroscope; (**c**) tilt sensing.

Then, according to Equations (6) and (7), we can determine the sensitivities of the gyroscope and accelerometer, respectively, as shown in Figure 9a,b. From Equation (11), the sensitivities of the tilt sensing can also be determined after eliminating the trend components, as shown in Figure 9c. The sensitivity frequency curves of the gyroscope, accelerometer, and tilt sensing have a similar trend, that is, the amplitude attenuation increases with the frequency, but the attenuation rates are different. The sensitivity attenuation of the gyroscope is only below 2.5 dB at a frequency of 2.5 Hz, while the attenuation of the accelerometer exceeds 4 dB at the same frequency. The sensitivity frequency curve of the tilt sensing is very close to that of the gyroscope, and seems to be unaffected by the sensitivity attenuation of the accelerometer. This is not difficult to understand, because the data from the accelerometer focus on remedying the drift rather than the amplitude. It can be seen that the sensitivity attenuation of the tilt sensing already exceeds 3.6 dB at a frequency of 3.15 Hz, which demonstrates that only 66% of the tilt can be sensed. Therefore, the MEMS inclinometer has a poor performance in tracking dynamic the tilts with a frequency of above 3.15 Hz.

### 4.2. Cross Coupling

**Figure 10:**
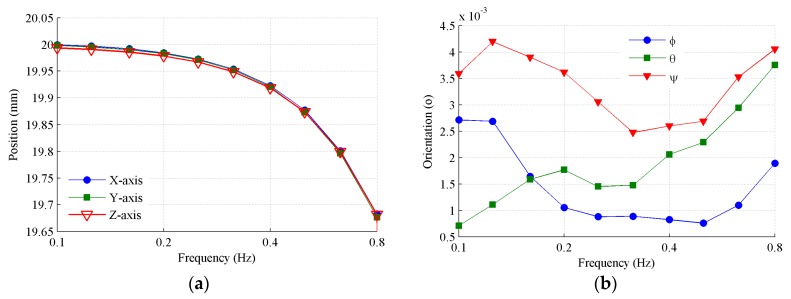
Positional orbit generated by the Stewart platform. (**a**) Amplitudes of position; (**b**) amplitudes of orientation in terms of Euler angles.

Furthermore, positional orbits from 0.1 to 0.8 Hz are generated as the spatial motion excitation, in order to test the cross coupling influence of the MEMS inclinometer. The desired amplitudes of the X-, Y-, and Z-axial positional vibration amplitudes are all set to 20 mm. Figure 10 shows the amplitudes of the orientation and position that are solved from the measured encoders. Figure 10a shows that a slight amplitude attenuation occurs as the frequency increases from 0.1 to 0.8 Hz. Figure 10b shows that the amplitudes of the orientation angle are less than 0.005° over the whole frequency range, so we can neglect their influence.

**Figure 11:**
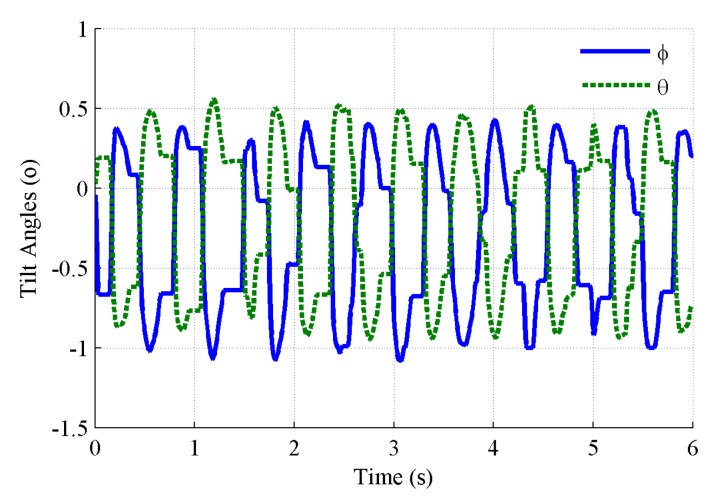
Tilt angles vary with time, caused by cross-coupling.

**Figure 12:**
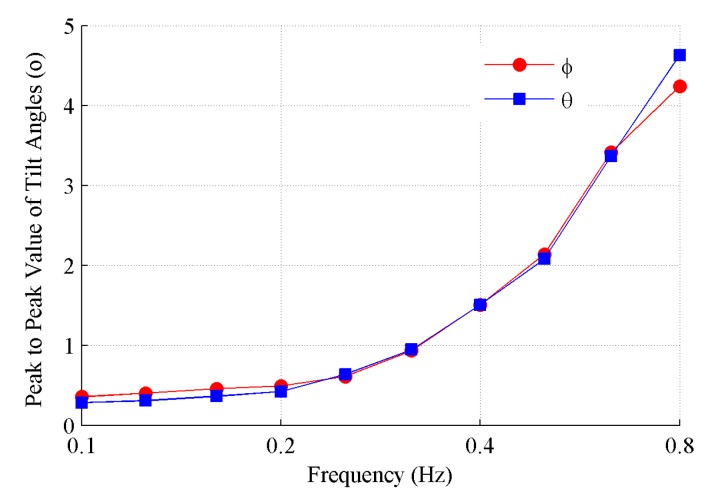
Peak-to-peak values of the tilt angles caused by cross-coupling.

The outputs of the tilt angles at the positional orbit with 0.4 Hz are depicted in Figure 11. It can be seen that both the roll and pitch angles are periodically repeated signals that are not ideal simple harmonic signals, because of the complex data fusion algorithm implemented. The repeated frequency is identical to that of the positional vibrations. The maximum and minimum deviations of the tilt angles are 0.5° and 1.0°, respectively. Figure 12 shows that the peak to peak values of the tilt angles increase with the frequency. This is easy to understand, because the acceleration disturbance caused by the positional vibration with a certain amplitude of displacement increases as the frequency increases. The change trend of the roll and pitch angles is very close and the peak-to-peak value of the tilt angles exceeds 2° at frequencies above 0.5 Hz. The MEMS inclinometer has an obvious cross-coupling influence because of the positional vibration with frequencies of above 0.5 Hz. For the sake of the accurate dynamic tilt measurement, the MEMS inclinometer should be mounted without a predominant positional vibration.

### 4.3. Comparison with the Rotator

**Figure 13:**
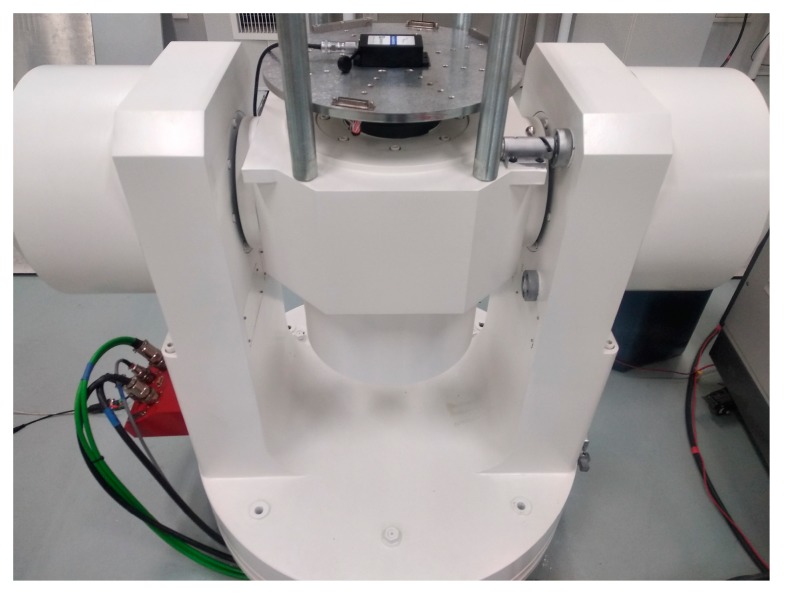
Two-axis rotator for MEMS inclinometer testing.

An experiment comparison with the rotator was conducted in order to verify the effectiveness of the proposed method. The two-axis rotator was employed to sequentially generate a single tilt motion. As shown in Figure 13, the same MEMS inclinometer was mounted on the table of the rotator. The two axes of the rotator were firstly positioned so that the roll axis of the inclinometer was parallel with the outer axis of the rotator. By controlling the outer axis to angularly vibrate, the roll-axis sensitivities of the gyroscope and tilt sensing were determined. Then, the pitch axis of the inclinometer was tuned to be parallel with the outer axis of the rotator, by which the pitch-axis sensitivities of the gyroscope and tilt sensing were determined.

**Figure 14:**
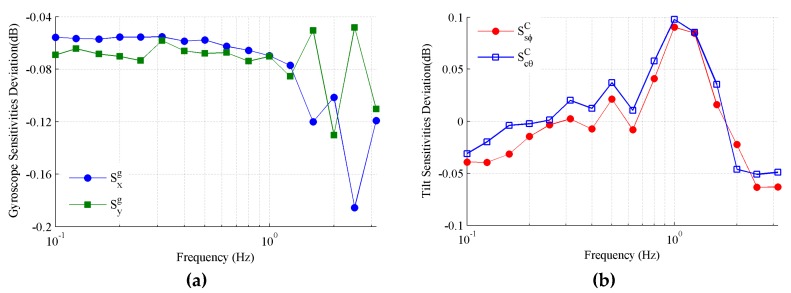
Amplitude frequency deviation between the Stewart platform and rotator. (**a**) Gyroscope; (**b**) tilt sensing.

As illustrated in Figure 14, the tested amplitude frequency deviations of the gyroscope sensitivities, and the tilt sensing sensitivities between the Stewart platform and the traditional rotator were less than 0.2 dB and 0.1 dB, respectively. The results of the conical motion generated by the Stewart platform match well with that of the uniaxial rotation generated by the rotator, which verifies the effectiveness of the proposed method.

However, the Stewart platform has an advantage over the uniaxial rotation device in dynamic tilt testing because of the following facts: (1) the generated conical motion provides two degrees of freedom of tilt motion based on which the accelerometers, gyroscopes, and tilt sensing of the MEMS inclinometer can be tested by just a single motion orbit, without being remounted; (2) the tip of the conical motion that is generated by the Stewart platform can be set flexibly instead of being fixed, as generated by the multi-axis rotator; (3) spatial positional orbits can also be generated for testing the cross coupling influence of the MEMS inclinometer.

## 5. Conclusions

We studied a new approach for the use of the Stewart platform to generate spatial orbits for testing a MEMS dynamic inclinometer. The results obtained from this study are summarized as follows:

The six limb lengths were calculated from the desired spatial orbits by implementing inverse kinematics, and were regarded as references to control the servo motors. The actual position and orientation of the moving platform were obtained from the six measured limb lengths by implementing forward kinematics.

Conical motion around the Z-axis, which provides two degrees of freedom of the dynamic tilt motion was generated for the dynamic tilt testing. To be unaffected by the rotation order of the orientation angles, the sensitivities of the tilt sensing in terms of the quaternion were defined. The sensitivities of the gyroscope, the accelerometer, and the tilt sensing were determined.

The experiment showed that the tested amplitude frequency deviations of the gyroscope sensitivities, and the tilt sensing sensitivities between the Stewart platform and the traditional rotator were less than 0.2 dB and 0.1 dB, respectively. The proposed method was verified to test the tracking performance and cross coupling of the MEMS inclinometer.

## Figures and Tables

**Table 1:** Micro-electro-mechanical system (MEMS) inclinometer characteristics.

Dynamic Accuracy	0.1°	Tilt Range	Pitch ± 90°, Roll ± 180°
Static accuracy	0.01°	Start delay	<50 ms
Resolution	0.01°	Maximum output frequency	100 Hz

## References

[1] Shaeffer D.K. (2013). MEMS inertial sensors: A tutorial overview. IEEE Commun. Mag..

[2] Bedon C., Bergamo E., Izzi M., Noè S. (2018). Prototyping and validation of MEMS accelerometers for structural health monitoring-the case study of the Pietratagliata cable-stayed bridge. J. Sens. Actuator Netw..

[3] Ha D., Park H., Choi S., Kim Y. (2013). A wireless MEMS-based inclinometer sensor node for structural health monitoring. Sensors.

[4] Ha D.W., Kim J.M., Kim Y., Park H.S. (2018). Development and application of a wireless MEMS-based borehole inclinometer for automated measurement of ground movement. Automat. Constr..

[5] Member S.N., Touya Y., Nonmembers S.T. (2010). Automatic on-line measurement of ship’s attitude by use of servo-type inclinometers. Electr. Commun. Jpn..

[6] Akella M.R., Halbert J.T., Kotamraju G.R. (2003). Rigid body attitude control with inclinometer and low-cost gyro measurements. Syst. Control Lett..

[7] Gui P., Tang L., Mukhopadhyay S. MEMS based IMU for tilting measurement: Comparison of complementary and kalman filter based data fusion. Proceedings of the 2015 IEEE 10th conference on Industrial Electronics and Applications (ICIEA).

[8] Ligorio G., Sabatini A.M. (2015). A Novel Kalman filter for human motion tracking with an inertial-based dynamic inclinometer. IEEE Trans. Biomed. Eng..

[9] Syed Z.F., Aggarwal P., Goodall C., Niu X., El-Sheimy N. (2007). A new multi-position calibration method for MEMS inertial navigation systems. Meas. Sci. Technol..

[10] Aggarwal P., Syed Z., Niu X., El-Sheimy N. (2008). A standard testing and calibration procedure for low cost MEMS inertial sensors and units. J. Navig..

[11] Ren Y., Wang Y., Wang M., Wu S., Wei B. (2014). A measuring system for well logging attitude and a method of sensor calibration. Sensors.

[12] Cheuk C.M., Lau T.K., Lin K.W., Liu Y. Automatic calibration for inertial measurement unit. Proceedings of the 2012 12th International Conference on Control Automation Robotics & Vision (ICARCV).

[13] Ren C., Liu Q., Fu T. (2015). A novel self-calibration method for MIMU. IEEE Sens. J..

[14] Fang B., Chou W., Ding L. (2014). An optimal calibration method for a MEMS inertial measurement unit. Int. J. Adv. Robot Syst..

[15] Gallardo-Alvarado J. (2016). An overview of parallel manipulators. Kinematic Analysis of Parallel Manipulators by Algebraic Screw Theory.

[16] Liu K., Fitzgerald J.M., Lewis F.L. (1993). Kinematic analysis of a Stewart platform manipulator. IEEE Trans. Ind. Electron..

[17] Furqan M., Suhaib M., Ahmad N. (2017). Studies on Stewart platform manipulator: A review. J. Mech. Sci. Technol..

[18] Cardona M. Kinematics and Jacobian analysis of a 6UPS Stewart-Gough platform. Proceedings of the 2016 IEEE 36th Central American and Panama Convention (CONCAPAN XXXVI).

[19] Zhang Y., Yu Y. Optimal design of 6DOF parallel robot based on output frequency response function. Proceedings of the 2009 International Conference on Measuring Technology and Mechatronics Automation.

[20] Jiang H.Z., He J.F., Tong Z.Z. (2010). Characteristics analysis of joint space inverse mass matrix for the optimal design of a 6-DOF parallel manipulator. Mech. Mach. Theory.

[21] Wu J.F., Zhang R., Wang R.H., Yao Y.X. (2014). A system optimization approach for the calibration of parallel kinematics machine tools by a laser tracker. Int. J. Mach. Tools Manuf..

[22] Ji P., Wu H. (2002). A closed-form forward kinematics solution for the 6-6/sup p/Stewart platform. IEEE T. Robotic. Autom..

[23] Pedley M. (2013). Tilt sensing using a three-axis accelerometer. Freescale Semicond. Appl. Notes.

[24] Liu Z., Cai C., Yu M., Yang M. (2017). Applying spatial orbit motion to accelerometer sensitivity measurement. IEEE Sens. J..

